# Individual consistency in the localised foraging behaviour of shy albatross (*Thalassarche cauta*)

**DOI:** 10.1002/ece3.10644

**Published:** 2023-10-24

**Authors:** Claire Mason, Alistair J. Hobday, Mary‐Anne Lea, Rachael Alderman

**Affiliations:** ^1^ Institute for Marine and Antarctic Studies University of Tasmania Battery Point Tasmania Australia; ^2^ CSIRO Environment Battery Point Tasmania Australia; ^3^ Department of Natural Resources and Environment Tasmanian Government Tasmania Hobart Australia

**Keywords:** foraging strategy, GPS telemetry, individual specialisation, seabirds, site fidelity

## Abstract

Quantifying the intra‐ and interindividual variation that exists within a population can provide meaningful insights into a population's vulnerability and response to rapid environmental change. We characterise the foraging behaviour of 308 trips taken by 96 shy albatross (*Thalassarche cauta*) from Albatross Island across seven consecutive years. At a population level, incubating shy albatross exploited a consistent area within ca. 500 km radius of their breeding colony. During half of the trips, individuals utilised the closest shelf break to the west of the colony, where upwelling events have been reported. The other half of the trips were exclusively within the neritic zone, utilising a variety of locations within the Bass Strait. Furthermore, we found evidence of individual consistency to geographic locations, with subsequent trips by an individual more similar than random trips from all individuals in our data, both within and between years (*G*‐test, *p* < .05). Between‐individual variation in foraging behaviour was not meaningfully explained by age (linear regression, *p* > .05) or sex (*t*‐test, *p* > .05) for any metric, suggesting that other intrinsic individual factors are accounting for between‐individual variation in foraging trips. A localised foraging distribution is unusual for albatross, which, combined with high variation in space use between individuals demonstrated here, suggests that this species is accessing adequate resources near the colony. Overall, these findings suggest that incubating shy albatross from Albatross Island exhibit tendencies of a generalist population comprised of uniquely specialised individuals. These results suggest that this species is operating below its biological capacity in this fast‐warming area and provide a baseline from which to assess future change.

## Graphical abstract

Using seven consecutive years of GPS telemetry data, we characterise the variation in foraging behaviour between and within shy albatross individuals from Albatross Island to assess their capacity to respond to changes in their marine environment. At a population‐level, shy albatross displayed a highly localised foraging distribution. However, we found variable space use between individuals, but consistency of space use within individuals. As a high‐trophic species in a climate change hotspot, a comprehensive description of behavioural tendencies adds to a growing understanding of shy albatross ecology and provides insights into the current and future impacts of climate change.

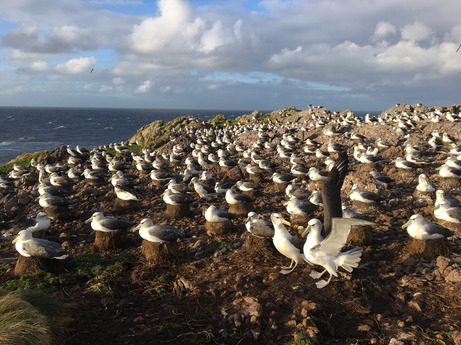

## 1 INTRODUCTION

Populations are comprised of individuals using a variety of narrower subsets of the populations' ecological niche (Bolnick et al., 2003). When conspecifics consistently vary in resource use regardless of age, sex, or phenology, it is termed ‘individual specialisation’ (Araújo et al., 2011; Bolnick et al., 2003). Overall, populations can be described as (A) similarly specialised individuals resulting in a specialist population, (B) distinctly specialised individuals resulting in a generalist population, or (C) generalist individuals resulting in a generalist population (Phillips et al., 2017). Variation in niche use between individuals (generalist population type B or C) can minimise intraspecific competition for resources within the population and, depending on the scale of habitats or resources used, can give populations resilience to environmental change through divergent responses (Edelaar & Bolnick, 2019). Populations comprised of similarly specialised individuals (specialist population type A) are theoretically less resilient to change (Clavel et al., 2011; Colles et al., 2009). Thus, quantifying the variability present within a population or species can shed light on their resilience or sensitivity to current and predicted environmental changes.

Foraging behaviour plays a key role in determining niche use (Carneiro et al., 2017) and characterising a species' sensitivity to climate change. Foraging decisions may be driven by environmental (Bost et al., 2015; Horswill et al., 2017), social (Urmy, 2021), or individual (Patrick & Weimerskirch, 2014; Votier et al., 2017) cues and constraints and may change with age (Patrick et al., 2020; Votier et al., 2017). Spatially, an individual may consistently forage in a preferred area which could increase local knowledge and experience, leading to increased foraging efficiency (Arthur et al., 2015; Call et al., 2008; Patrick & Weimerskirch, 2014). However, if the chosen spot is not consistently productive, fidelity might be more costly than exploratory foraging decisions (Eliassen et al., 2007), such as behavioural models that assume individuals make decisions based on the previous success of foraging (e.g. ‘foraging by expectation’ (Piatt, 1990) or ‘win‐stay‐lose‐shift’ (Bonnet‐Lebrun et al., 2021)). The similarity of foraging decisions between individuals can indicate population‐level sensitivity to climate change. Individuals making similar decisions or following social cues may be able to share beneficial foraging information and navigational cues (Urmy, 2021); however, a potential disadvantage could be an increase in competition for resources (Sherley et al., 2017). Alternatively, a population consisting of similarly specialised individuals could indicate strong environmental or biological constraints (Ceia & Ramos, 2015).

There is strong and growing evidence of the impacts of climate change on seabirds, particularly changes in oceanographic processes causing shifts in the distribution and availability of prey (Dias et al., 2019). Sensitivity to these rapid changes is, in part, due to their life history strategy (i.e. long‐lived, delayed maturity, and low fecundity) limiting the capacity for rapid evolutionary adaptation. Natal philopatry and central place foraging behaviour also add to climate change vulnerability, requiring productive oceanic areas accessible from static breeding sites. Climate change effects also compound decades of anthropogenic pressures threatening seabird populations, such as harvesting, fisheries interactions, habitat loss, and marine pollution (Dias et al., 2019).

While many albatross species make circumpolar navigations (Butcher et al., 1997; Croxall et al., 2005; Thompson et al., 2021), shy albatross (*Thalassarche cauta*; Robertson & Nunn, 1998) remain at one of three colonies and in surrounding southern Australian waters almost year‐round (Hedd & Gales, 2005). Their restricted range is speculated to be linked to the predictably reliable resources in proximity to the colony (Hedd & Gales, 2005). Additional support for this hypothesis includes a year‐round presence at the colony, an unusually short pre‐lay foraging exodus (Abbott et al., 2006), shorter incubation shifts (Hedd & Gales, 2005), and the highest recorded frequency of chick feeding compared to similar albatross species (Hedd et al., 2002). From these observations, shy albatross are not exhibiting extreme foraging behaviours for the Diomedeidae family. Thus, the shy albatross, compared to other species in the Diomedeidae family, could have a greater capacity to increase investment in longer or further foraging trips if pressures increase such as local resources are not adequately productive (Dufour et al., 2021; Hedd & Gales, 2005). However, underlying this hypothesis is the assumption shy albatross have the flexibility to adapt to changes that arise, for example, shifts in prey distribution and availability.

South‐eastern Australia is warming at about four times the global average and is considered a ‘climate change hotspot’ (Hobday & Pecl, 2014; IPCC, 2021). Substantial impacts of this warming have already occurred in marine ecosystems such as rapid range shifts for many species (Champion et al., 2018; Gervais et al., 2021), dieback of kelp forest ecosystems (Butler et al., 2020), and marine heatwaves (Oliver et al., 2017). In our study region of southeast Australia, two significant marine heatwaves occurred during the time series of our data including a strong, long‐lasting heatwave in Tasmanian waters in 2015/16 (Oliver et al., 2017) and a strong but brief heatwave in 2017 (Oliver et al., 2021). We set out to characterise the population‐ and individual‐level variability in foraging behaviour inferred from GPS telemetry tags deployed on adult birds. The rationale for this research is to provide insights into the resilience of this population to climate change. In addition, we also investigate potential contributing factors to individual consistency and foraging behaviour and the potential implications under climate change, and so explore age, sex, evidence for learning, and the extrinsic factor of bathymetry as part of our analysis.

The GPS telemetry dataset spans seven consecutive seasons, comprising 139 separate deployments, across 96 individual shy albatross from the Albatross Island colony. This dataset enables the calculation of foraging traits and tendencies (such as trip duration and consistency) to determine intra‐ and interindividual variation in the shy albatross population. As well as characterising the foraging behaviour and space use for the population, we explore individual consistency of space use and the directness of subsequent trips by an individual. We test for evidence of generalist or specialist tendencies of individuals and hence the population as a whole (Phillips et al., 2017). We assume that a population containing greater variation between individuals could display divergent responses to environmental change, and hence, more resilience to climate change.

## 2 METHODS

### 2.1 Study site and field deployment

Albatross Island (40°22′38″ S, 144°39′20″ E) is a 18 ha island, 30 km off the northwest coast of Tasmania, Australia and is the annual breeding colony of ca. 5500 pairs of shy albatross (Alderman et al., 2011). Shy albatross lay eggs in September, chicks hatch in December, and juveniles fledge in late April. Shy albatross form monogamous, long‐term partnerships and equally share incubating and chick‐rearing duties (Abbott et al., 2006; Hedd et al., 2002).

Over seven consecutive years during the incubation stage, we deployed GPS tags on shy albatross from Albatross Island (Table 1). We attached CatLog Gen 2 GPS/GNSS Loggers (Perthold Engineering LLC) to mantle feathers using TESA tape and Loctite glue. Tags were shrink‐wrapped with heat in clear electrical tape for waterproofing. Tags weighed <30 g, which is 0.5% of the average ~6 kg adult shy albatross mass. Deployments occurred at nest sites that were monitored several times throughout the season as part of long‐term population monitoring. Known age, banded individuals were preferentially selected. We use adults in the incubation breeding stage for logistical ease and to reduce disturbance. Shy albatross are central place foragers during the entire breeding season; however, the incubation stage is when breeding individuals are the least constrained. During the chick brooding and chick‐rearing stages of the breeding season, individuals are required to provision chicks and foraging trips become more frequent and intensive (Hedd & Gales, 2005).

**TABLE 1:** GPS telemetry deployments during the incubation stage for shy albatross (*Thalassarche cauta*) from Albatross Island (40°22′38″ S, 144°39′20″ E) used in this study.

	*N* deployments	*N* trips captured	Max. deployment	Max. deployment (days)	Sampling resolution (min)
2013	52	61	24 Sept – 9 Oct	15	10
2014	15	17	23 Sept – 6 Oct	13	10
2015	15	19	23 Sept – 3 Oct	10	10
2016	15	19	7 Oct – 20 Nov	44	10
2017	15	26	29 Sep – 14 Nov	46	10
2018^b^	14	152	23 Aug – 5 Oct	44	20^a^
2019	13	14	29‐Sep – 12 Oct	13	10
TOTAL	139	308			

^a^ This deployment captured late‐winter foraging for another research question, so the temporal resolution of sampling was extended to ensure the longevity of battery life.

^b^ In 2018, trip metrics were calculated on incubation trips only (22 September onward), which included 14 deployments, 27 trips captured, and a maximum deployment length of 13 days.

### 2.2 Trip definition

Raw location estimates with timestamps were downloaded from retrieved tags using CatLog Control Centre software (Perthold Engineering). We calculated the (Haversine) distance between each location estimate and the centre of the colony (South study colony: longitude = 144.6557384, latitude = −40.3780953). This technology had an estimated accuracy from the manufacturer of 5–10 m; however, we observed displacement of up to 350 m for ‘nesting’ birds. Thus, we used 400 m displacement to categorise locations at the colony or at sea.

### 2.3 Data were cleaned and individual trips were created

Most trips could be automatically assigned; however, in a few cases, some trips did not start or end within the 400 m radius. We manually rectified these. In cases when a trip started outside the 400 m radius (i.e. started at sea), this was due to our field methods, as we deliberately chose birds whose partner had just returned to relieve them on the egg to reduce the risk of any disruption to incubation. Thus, on release, the tagged bird could immediately leave the island within the 10‐min window. Immediately commencing a foraging trip on the return of the partner is typical behaviour of many individuals coming off duty from incubation (C. Mason, personal observations). We manually flagged these trip starts. Our trip definition methods created 117 trips with ten or fewer location estimates, 79 of which were single‐location trips. All trips with ten or fewer location estimates were excluded from the dataset. Thirteen trips had maximum displacement greater than total distance, i.e. our metric ‘S’ > 0.5, due to trip starts and ends being first and last locations at sea rather than at the colony. These trips were removed. The final, cleaned data set, contained 308 distinct foraging trips from 139 device deployments from 96 unique individual birds (Table 1). A ‘trip’ is henceforth considered a distinct foraging trip, regardless of individual or deployment period, with greater than 10 location fixes away from the colony. There were 22 individuals tracked over multiple years and 43 individuals for whom we captured more than one trip per year.

From previous analyses of telemetry data collected during the incubation period from this population, a sample of 75 trips is sufficient to explain the spatial variation of 99% of the estimated population (Mason et al., 2018), thus we can be confident that our entire sample of 96 known unique individuals and over 300 trips is representative of the entire Albatross Island population of shy albatross during this time. At an annual scale (Table 1), our samples of 12–15 individuals capture 50% of the spatial variation explained by the population (Mason, 2016). Although this does not allow strong conclusions on how the entire population responded in a given year to the marine environment our samples are still substantial and informative, and so we explore some interannual questions with caution.

### 2.4 Trip metrics

In 2018, the deployment began in August to capture late winter foraging to address another research question. Although we retained the late‐winter data when exploring individual consistency, we excluded these late‐winter trips when calculating trip metrics as they are not representative of the foraging behaviour of adults during the incubation period.

To characterise a foraging trip, we extracted the coordinate of maximum displacement from the colony for each trip and the bearing from the colony to this point. We also calculated metrics describing the geometry of foraging trips including total distance, duration, and a measure of trip sinuosity (hereafter ‘S’) calculated by dividing maximum displacement by total distance. We classified the maximum displacement coordinate on foraging trips into three broad geographic areas: east (bearing 355–130°), northwest (bearing 260–355°), and southwest (bearing 130–260°) based on preliminary inspection of the data and the known importance of the shelf break for productivity in the region.

### 2.5 Individual consistency

We quantified the similarity of trips made by the same individual to identify the tendency for individual consistency within and across years. The proximity between the maximum displacement coordinates of two trips is used as a spatial similarity metric of trips for our purposes rather than the common approach of rasterising tracks into time spent squares (e.g. Alderman et al., 2010). In a time‐spent‐squares approach, neighbouring squares, although very similar spatially, have no association, therefore trips must be identical at the scale of the grid cell resolution to be considered ‘similar’ unless cells are very big. We used all trips, including late winter, for this analysis (*n* = 308). Although the data spanned the cusp of the breeding season, it allowed us to explore the similarity of consecutive trips by an individual. We used the maximum displacement coordinate to compare trip similarity. The distance between the maximum displacement coordinates for a pair of trips was calculated for (i) all possible pairwise combinations of trips in our data to create a ‘random’ reference level, (ii) all pairwise comparisons of trips captured during the same deployment for an individual, i.e. within season consistency, and (iii) all pairwise comparisons of trips taken by an individual that was tracked over multiple seasons, i.e. between season consistency. Our reference level distance matrix (i) between all trips in our data contained 4560 pairwise comparisons. For within‐season comparisons (ii) for each season, we created a distance matrix for all trips captured by a single deployment. A total of 75 pairs were generated. For between‐season comparisons (iii), a random trip was chosen for each individual in a season, and all pairwise comparisons were calculated using a distance matrix. A total of 91 pairs were identified from our dataset.

These within‐season (ii) and between‐season (iii) distance matrices were plotted and compared statistically to a frequency distribution for all pairwise trip comparisons in our dataset (i). This allowed us to test the hypothesis that trips by the same individual are more similar (using the maximum displacement coordinate) than trips by different individuals. A *G*‐test statistic was calculated for the two distributions to ascertain whether they differed statistically.

Assuming that more direct trips represent directed travel and a more informed or experienced individual, we compared the difference in trip sinuosity (‘S’) values across subsequent trips taken by an individual within a season as well as across subsequent seasons. This provides insights as to whether individual birds are learning over time.

### 2.6 Life history traits

The shy albatross population on Albatross Island has been monitored using a leg‐banding program since 1980. A section of the colony at the south end of the island has received intensive band resights and was the location of GPS telemetry deployments during this study. We obtained the breeding attempt status for each deployment (i.e. pre‐fledgling juvenile alive or failed breeding attempt at the end of the season). A total of 287 trips in our dataset were from banded birds, many with known ages and records of historical breeding history. Thus, age and sex could be assigned to most (92%) individual trips. Un‐banded birds made the remaining 21 (7%) trips. From our dataset of 96 unique bands, there were 32 genetically determined females and 30 genetically determined males (Australian Genome Research Facility). Five females and six males were assigned from observational morphology in the field (Hedd et al., 1998). There were 26 individuals with no sex assigned. Most trip metrics (6 out of 8 comparisons) had no statistical difference between genetically and morphologically identified males and females. The exceptions were the total distance travelled between genetically and morphometrically assigned male classifications (*T*‐test; *p* = .001) and ‘S’ between genetically and morphometrically assigned female classifications (*T*‐test; *p* = .002). Morphometrical sex determination has been successful for this species in the past (Hedd et al., 1998), so all morphological sex determination was retained, and the final data thus contained 73 birds (and 188 trips) with sex assigned. Age was assigned when the bird was banded as a chick (*n* = 68 individuals, 220 trips) and ranged from seven to 36 years.

We tested the null hypothesis that no trip metrics (bearing to maximum displacement coordinate, total distance, duration, and trip sinuosity) could be explained by sex (*t*‐test) or age (linear regression).

### 2.7 Bathymetry data

We used data hosted by the Australian Antarctic Division through the ‘raadtools’ R package. We extracted the seafloor depth below each location estimate from bathymetry data to compare the bathymetry encountered between different trips. We use the following three categories for classifying locations by their bathymetry, shelf: 0–200 m depth; shelf break: 200–1000 m depth, and pelagic: >1000 m depth. The oceanographic features created by the shelf break (upwelling, fronts) occur across the width of the shelf break (~2–5 km in this region); for our research questions, we consider all locations within this depth range (200–1000 m) as influenced by shelf break bathymetry.

Code in R programming language (R Core Team, 2022) and data to recreate all analyses are available from online repositories, please see data availability statement.

## 3 RESULTS

### 3.1 Population‐level patterns

During the incubation stage, shy albatross from Albatross Island exploited an area within ca. 500 km radius of their breeding colony, with 49% of the trips remaining within the neritic zone (the top ocean layer above the continental shelf). All other individuals encountered the shelf break during foraging trips, around half of these (24% overall) encountered shelf break bathymetry (200–1000 m depth) and the remaining half (27% overall) went beyond 1000 m depth into more pelagic waters. The deepest water encountered was 5092 m.

**FIGURE 1:**
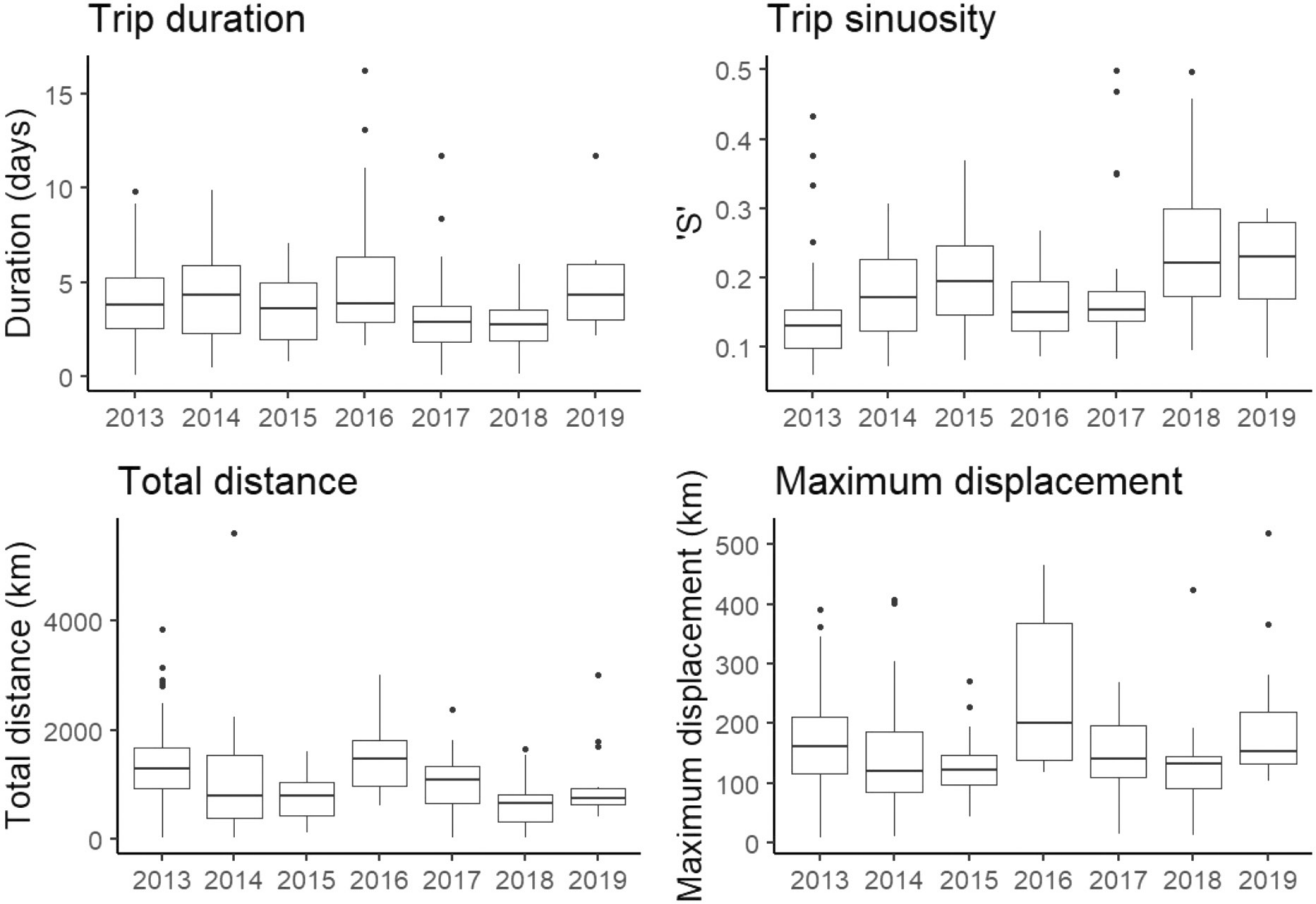
Foraging trip metrics across the seven years from Albatross Island shy albatross during the incubation stage.

Adults during the incubation stage took foraging trips between 2 h and 16 days in length, with an average of 3.9 days (Figure 1). Trips varied in maximum displacement from the colony between 9 and 520 km. Trip distance ranged between 23 and 5600 km, with an average of ~1100 km (Figure 1). The longest trip in our data set was 5600 km in 10 days, however, the bird remained within 400 km of the colony.

**FIGURE 2:**
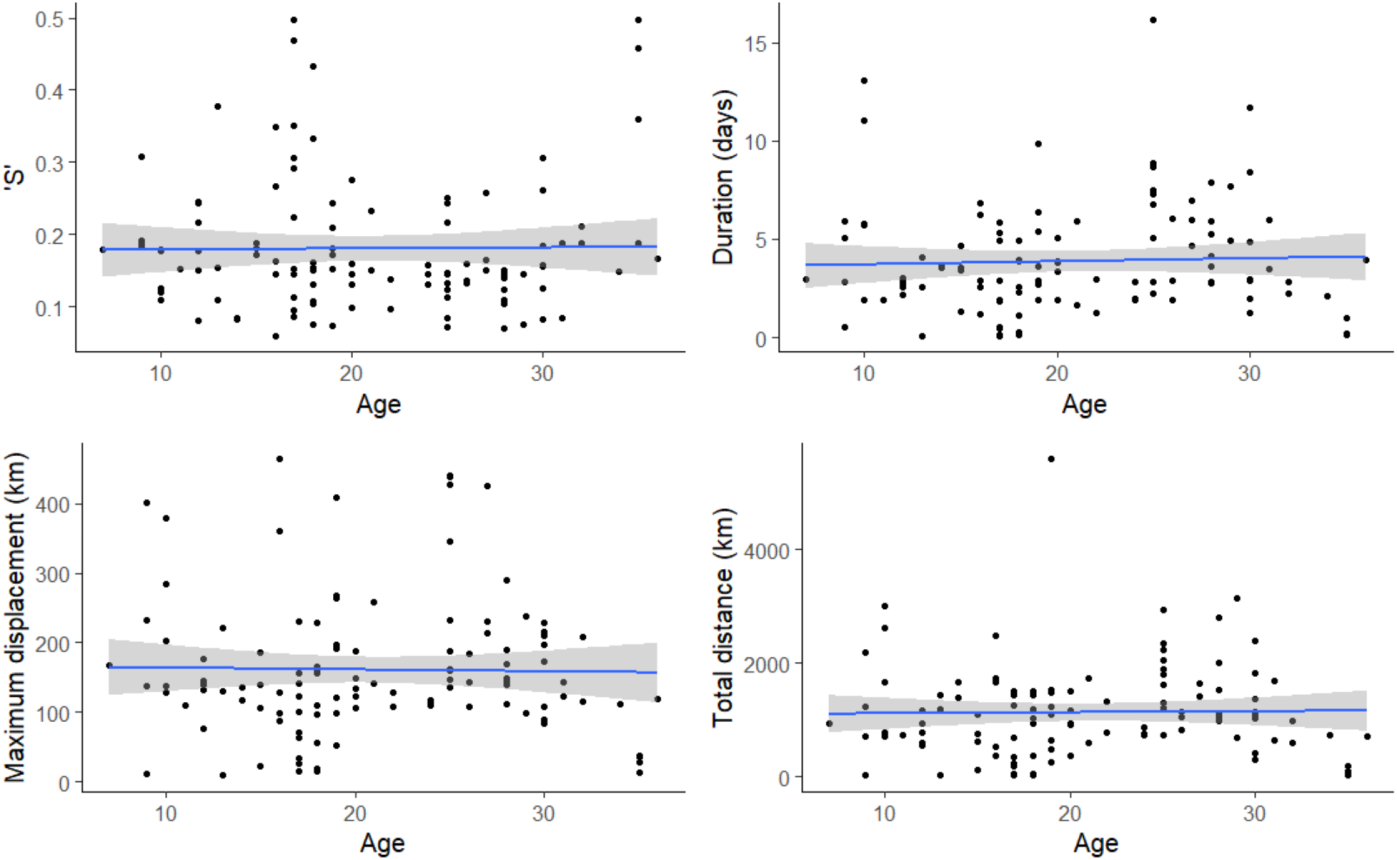
Relationship between four trip metrics and age for 115 trips made by known age incubating shy albatross from Albatross Island (filled circles). The straight line is the age estimated by linear regression, and the gray region depicts the 95% confidence interval for the mean.

The null hypothesis was supported by our data, that foraging behaviour was not significantly explained by age (linear regression, *p* > .05) or sex (*t*‐test, *p* > .05) for any metric (Figure 2).

**FIGURE 3:**
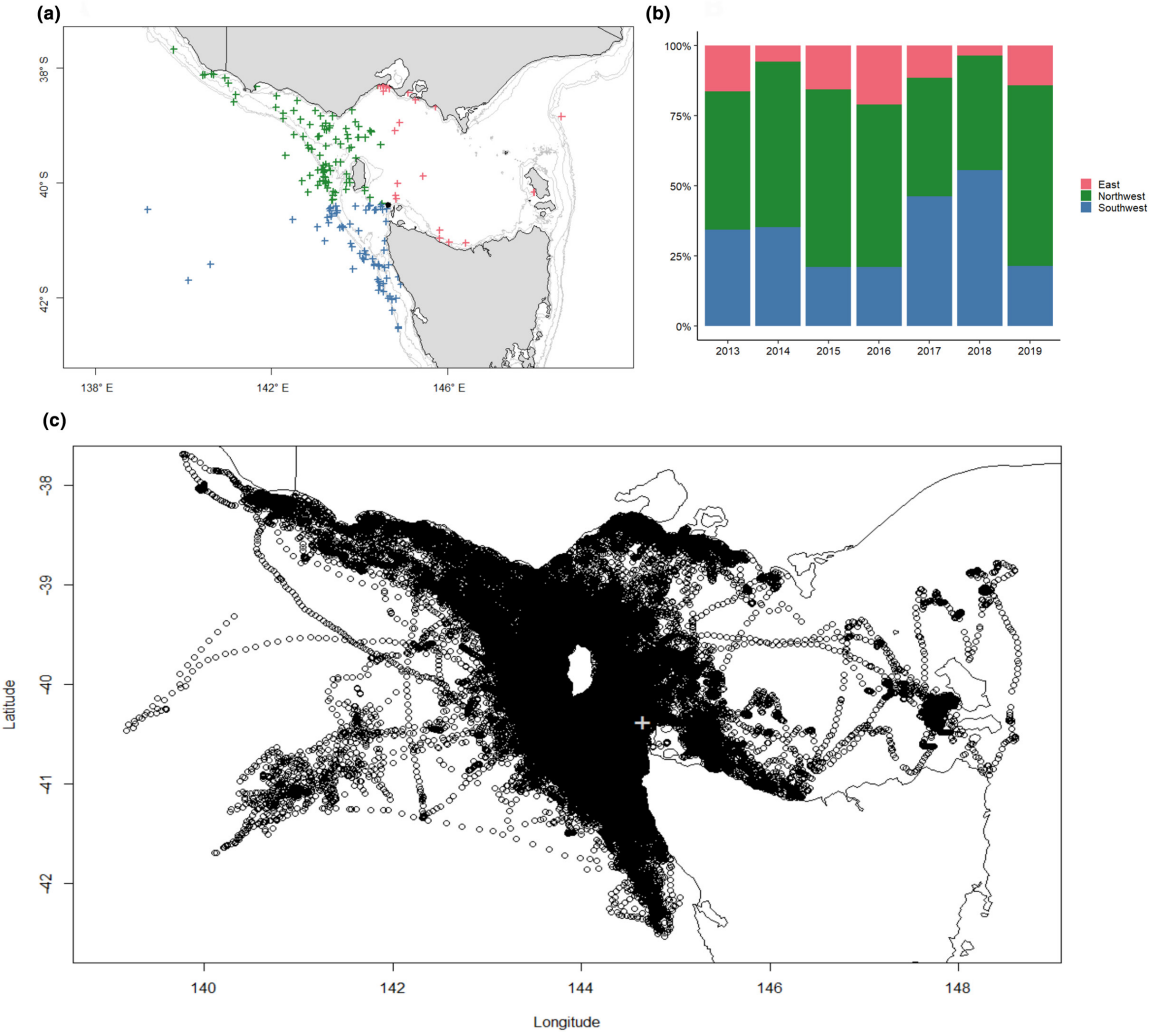
(a) Geographic regions visited during a foraging trip during the incubation period by shy albatross (*Thalassarche cauta*) from Albatross Island, categorisation based on the bearing between the colony and the maximum displacement coordinate for each trip. Light gray lines indicate bathymetry (20–400 m depth). (b) variation between seasonal deployments in the number of trips visiting each region. (c) all tracks captured by shy albatross adults during the incubation breeding period used in our study. The black filled circle in (a) and the gray cross in (c) depicts Albatross Island, the breeding colony.

Individuals predominantly travelled west from the colony (87% of maximum displacement coordinates) toward the closest shelf break (Figure 3). The average bearing across all seasons was west‐northwest (286°) and 44% of maximum displacement coordinates were within the northwest quadrant from the colony (270–360°; Figure 3). Average bearing did not significantly change between seasons (Circular analysis of variance LRT, ChiSq_6_ = 9.59, *p* = .14). Although the year was statistically significant in explaining trip duration (ANOVA; duration *F*_6,176_ = 3.08, *p* = .0068, Figure 2), as our sample size only allowed us to capture 50% of the spatial variation explained by the population, annual differences are more reflective of individual variation rather than the population as a whole during that year. A map of all foraging trips in full can be found in Figure 3c.

Our sample size for 2014–2019 was 12–15 individuals and only able to capture 50% of the spatial variation explained by the population (Mason, 2016), thus, although year had a significant relationship with all trip metrics (ANOVA; duration *F*_6,176_ = 3.08, *p* = .0068; sinuosity *F*_6,176_ = 5.62, *p* = 2e‐5; total distance *F*_6,176_ = 5.38, *p* = 4e‐5; maximum displacement *F*_6,176_ = 4.79, *p* = .00015), the annual differences are more reflective of the variation between samples rather than the population as a whole during that year (Figure 1).

The data contained 69 known‐age individuals (115 trips) who were banded as chicks. The relationship between all trip metrics and age was statistically insignificant (total distance *F*_1,112_ = 0.03, *p* = .86; maximum displacement *F*_1,112_ = 0.04, *p* = .84; sinuosity *F*_1,112_ = 0.01, *p* = .91; duration *F*_1,112_ = 0.18, *p* = .67), and there was no visible trend (Figure 2).

### 3.2 Individual‐level patterns

**FIGURE 4:**
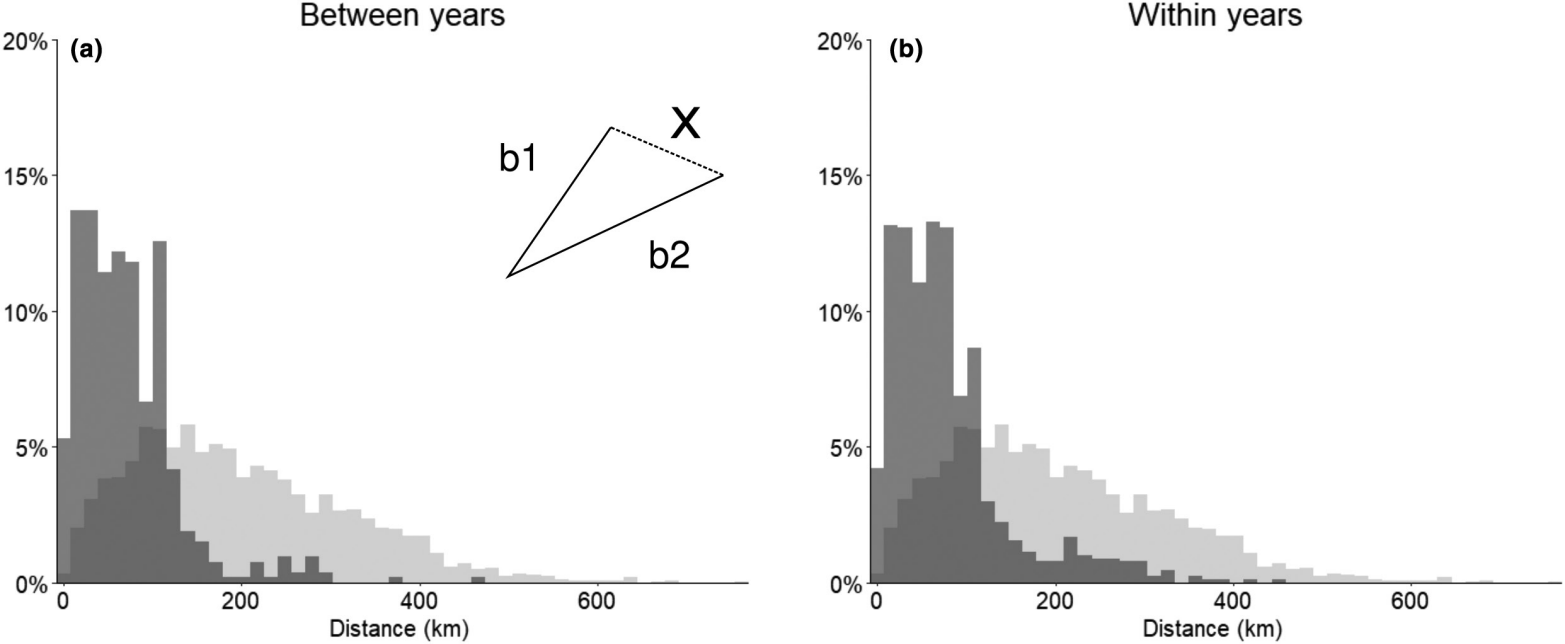
Foraging trip metric quantifying repeat use of space by individuals (a) between and (b) within years. Frequency distribution of the distance (X) between maximum displacement coordinates of two trips (b1 and b2) by the same bird (dark; (a) between years, *n* = 91, (b) within years, *n* = 75) or by two random individuals (light; *n* = 4560 pairs).

**FIGURE 5:**
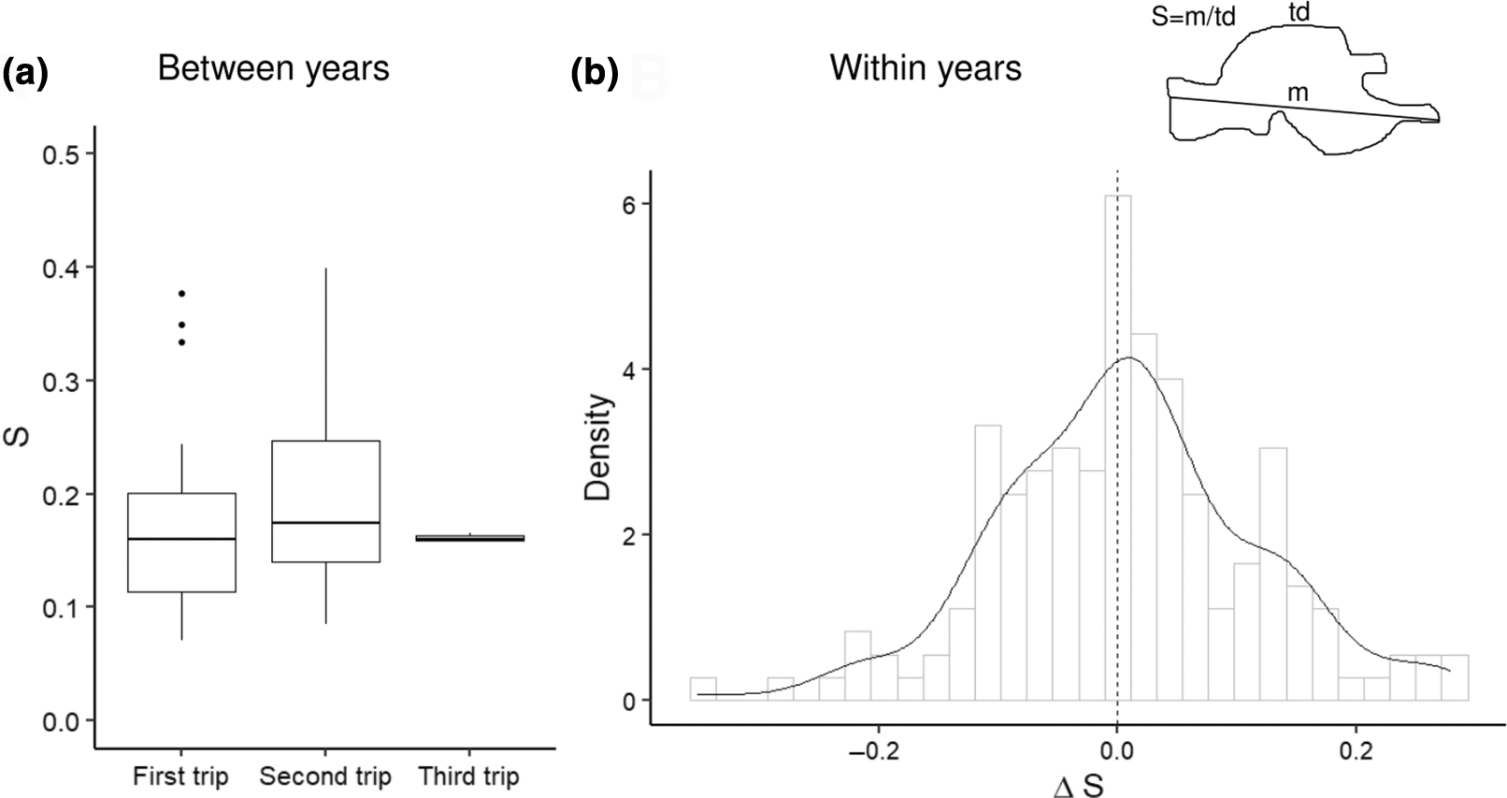
The difference in trip sinuosity (‘S’ = m/td; m: maximum distance, td: total distance) on subsequent trips by the same shy albatross. (a) Between years when the same individual was tagged over multiple years (first and second trips each *n* = 22, third *n* = 3) and (b) within a year when a deployment captured multiple trips.

We quantified the similarity between pairs of trips taken by the same individual to trips taken by random pairs. The distance between the maximum coordinates was statistically closer for trips taken by the same individual than for the distances between random trips within and between years (*G* test *p* < .05, Figure 4). There was no increase in trip directness (decrease in ‘S’) on subsequent trips by the same individual, either within or between years (Figure 5).

### 3.3 Life history drivers of behaviour

There was no evidence that age had an effect on trip metrics (linear regression, *p* > .05; Figure 2). Sex or breeding attempt status did not have a significant influence on any trip metrics in our data set (*T*‐test, *p* > .05). Although no trip metrics for sex were significantly different, the mean bearing for trips taken by males was statistically more southerly than female trips (Circular Analysis of Variance LRT, ChiSq_1_ = 21.91, *p* < .0001).

## 4 DISCUSSION

Our motivation was to provide insights into the sensitivity of shy albatross to climate change, by testing for evidence of generalist or specialist foraging behaviour tendencies (Phillips et al., 2017). Our data revealed shy albatross from Albatross Island used a foraging area within 500 km of the breeding colony during the incubation stage. This is a small range for a taxonomic family renowned for vast ocean movements. This suggests that the area around the Albatross Island colony contains adequate resources for the population during the breeding season. Given the overwintering presence of birds on the island, this might also be the case year‐round (Hedd & Gales, 2005). On an individual level, however, we found evidence of individuals visiting locations that were closer to each other than random locations both within and between years, suggesting individual consistency (a proxy for individual specialisation) to foraging areas exists in the population. Overall, these findings suggest that during the incubation stage, shy albatross from Albatross Island exhibit tendencies of a generalist population comprised of uniquely specialised individuals (Phillips et al., 2017).

### 4.1 Evidence of individual consistency

Although optimal foraging strategies at a population level can be estimated using optimal foraging theory, many wild populations demonstrate a large degree of consistent individual variability, i.e. individual specialisation (Bolnick et al., 2003). Foraging specialisations are thought to arise when predictable and diverse resources exist to minimise competition by divergent niches. Individualised behaviour is thought to be learned during ontogeny through experiences had by individuals (Collet et al., 2020; Orgeret et al., 2019). Although innate drivers such as age, sex, or morphology, and more recently personality (Harris et al., 2020; Patrick et al., 2017) can explain a proportion of variation in habitat use (Weimerskirch et al., 2014), individual consistency in foraging behaviour can also be unexplained by any of these factors (Bolnick et al., 2003; Woo et al., 2008). The factors we included in our study (age and sex) were not related to the variation we observed.

Within seasons, over the same ~1–2 weeks, our data show individuals departing the colony within hours of each other accessing a variety of areas of the Bass Strait region (Figure 3), suggesting that environmental cues are not key in shaping foraging decisions in the same way for all individuals in this population. Limited overlap between individuals, and the use of distinct regions of the Bass Strait that we observed, was found previously in a study of five shy albatross (Hedd et al., 2001). In all seven seasons of our study, individuals used many areas of the Bass Strait (Figure 3b), suggesting that on a population level, all regions are important regardless of year, providing further support for the concept that individual preference is driving the areas used by an individual, rather than all birds (or individuals from age classes or sex) responding to cues in the same way.

Our data show individuals visiting similar locations during the same year (Figure 4a), and in subsequent years (Figure 4b), to a greater degree than expected by chance. However, we did not find an increase in trip directness over time that would suggest learning (Figure 5). This finding suggests that shy albatross display a high degree of individual preference for geographic locations, and this preference might be established early in a bird's life. A previous tracking study of breeding shy albatross found some evidence of site fidelity within years and between years throughout the breeding season, but not complete overlap (Hedd et al., 2001). The repeated use of an area has been experimentally and empirically shown to be linked to the profitability of the previous trip to that spot (Bonnet‐Lebrun et al., 2021). This strategy has been termed the ‘win‐stay‐lose‐shift’ strategy. Previous work has shown that shy albatross juveniles from Albatross Island show a greater tendency to re‐use the same grid cells on different foraging trips than juveniles from the other two breeding colonies and have greater survival outcomes (Alderman et al., 2010). Thus, the presence of individual consistency by shy albatross in our data suggests birds are experiencing profitable foraging trips (e.g. Lima, 1983). For long‐lived species, such as shy albatross, although the profitability of a chosen site likely varies and might not be maximal in all years, over longer time scales (shy albatross can live for more than 30 years), net productivity could be maximised using this strategy (Arthur et al., 2015; Bradshaw et al., 2004).

Age did not statistically explain variation in foraging behaviour using our metrics (Figure 2), and we found no evidence of subsequent trips by an individual becoming more direct, either by duration or reduced trip sinuosity, within or between years (Figure 5). The foraging behaviour of breeding shy albatross did not capture any evidence of individuals improving their foraging efficiency with age (Figure 5), as has been demonstrated in other systems (Eliassen et al., 2007), or did not provide an understanding of ontogeny‐driven foraging behaviour that has been explored in the extreme movement exhibited by other long‐lived seabirds (Collet et al., 2020; Corbeau et al., 2020; Orgeret et al., 2019). Our finding that males were using more southerly locations was potentially due to bias in the data arising from earlier deployments in August 2018, where the majority of tagged birds were males. Furthermore, foraging behaviour has been shown to vary between breeding stages for shy albatross (Hedd et al., 2001), so our finding of individual consistency may not hold throughout the breeding season. Additional tracks across the entire year are needed to address this consistency question.

### 4.2 The Bass Strait as the shy albatross backyard

A widely held assumption is that resources in the ocean are unpredictable and patchy (Ritz et al., 2011), which drives the extreme life history traits exhibited in the Diomedeidae family (Weimerskirch, 2007). However, empirical evidence for the patchy distribution of albatross prey species is often lacking, especially in the neritic Bass Strait (Huang & Wang, 2019; Nieblas et al., 2009; Sandery & Kämpf, 2007). Our findings provide further support for the hypothesis that the restricted foraging behaviour of shy albatross is in response to a reliable and sufficient supply of local resources for their annual breeding cycle (Piatt et al., 2006; Yen et al., 2004).

Fifty‐one percent of trips exploited the shelf break in the western Bass Strait – a static bathymetric feature west of the breeding colony that has been known as a shy albatross foraging region (Brothers et al., 1998; Hedd et al., 2001). A reoccurring spring phytoplankton bloom has been reported off the west coast of Tasmania (Kämpf, 2015), overlapping temporally and spatially with our study. In addition to the spring event, sporadic, year‐round upwelling events have been shown to occur (Kämpf, 2015), perhaps providing resources for the Albatross Island population throughout the summer breeding cycle near the colony. Furthermore, the closely related white‐capped albatross (*Thalassarche steadi*;   Robertson & Nunn, 1998) also uses the Bass Strait during its breeding season (beginning in December, two months later than shy albatross), travelling more than 2000 km from breeding colonies in *Aotearoa*/New Zealand (David Thompson NIWA, unpublished data).

Shy albatross diet consists of predominantly fish and cephalopods, with ~100 prey species detected over a four‐year study (McInnes et al., 2020). The most distinct stage in their diet is during incubation, where ~20–50% of shy albatross diet is comprised of *Sepia apama* (Australian giant cuttlefish, McInnes et al., 2020). The only documented mass spawning aggregation of *S. apama* occurs in the Spencer Gulf in South Australia, ca. 1000 km northwest of the breeding colony of shy albatross in our study (Hall & Hanlon, 2002). The peak of this aggregation is in May and concludes with mass terminal spawning, with floating moribund or deceased cuttlefish accessible to shy albatross. Our results show that shy albatross remain within 500 km of the breeding colony during the early breeding period, which is also around four months after the peak of the South Australian aggregation. Thus, the Spencer Gulf cephalopod aggregation may not be the source of *S. apama* in the diet of shy albatross from Albatross Island during the incubation period. Anecdotal reports (S. Ling, unpublished) of an *S. apama* spawning aggregation in Bass Strait closer to the shy albatross incubation stage could explain the large component of *S. apama* in the shy albatross diet.

### 4.3 Do these data suggest resilience to climate change?

We aimed to examine intra‐ and interannual variation in shy albatross foraging behaviour to understand the potential for flexibility (Pecl et al., 2014) across a relatively short period (7 years). From previous population representation analysis, the annual trip metrics from a sample of ~15 individuals explain at least 50% of the variation exhibited by the population (Mason, 2016; Mason et al., 2018). Although an analysis of variance for all metrics between years was statistically significant, biologically, there is high interannual consistency in trip metrics (Figure 1), especially given the two extreme heatwave events that occurred in this region over our study period (i.e. in 2015/16 (Oliver et al., 2017) and in 2017 (Oliver et al., 2021)).

This study only captures foraging behaviour from Albatross Island, the northern population of shy albatross. The two southerly colonies, including the largest population on The Mewstone, have been shown to have distinct post‐fledgling dispersal patterns compared to the Albatross Island population (Alderman et al., 2010), but adults are thought to also have a localised range (Brothers et al., 1998). Comprehensive tracking of the distribution of these southerly colonies during the breeding season is needed before species patterns can be elucidated.

Although shy albatross possess traits that suggest sensitivity to climate change (i.e. only three populations, K‐selected life history strategy), it is complex to predict what the impacts of current and future climate change will be. Our large, high‐resolution dataset, which comprehensively describes the foraging behaviour and distribution of adults during the incubation stage, revealed the presence of individual consistency to geographic areas, a proxy for individual specialisation. On a population level, shy albatross exhibited generalist tendencies with individuals in the same deployment using a variety of areas, however, all within the localised distribution of the western Bass Strait and shelf break. We can speculate that at the population level, their ability to modify foraging distribution is not constrained and birds have the capacity to move further afield, and thus, could have inbuilt flexibility to cope with change, making them less vulnerable to climate change. However, broad‐scale climate change impacts across southern Australia will likely impact productivity for all predators in southern Australia (Sydeman et al., 2015).

At a larger scale, southeastern Australian waters are considered a ‘climate change hotspot’, warming at about four times the global average (Hobday & Pecl, 2014; IPCC, 2021), driven, in part, by the southern extension of the East Australian Boundary Current (Ridgway, 2007a, 2007b). Thus, shy albatross are and will experience the impacts of climate change sooner than some other regions of the world. Coastal upwelling events that generate productivity in southern Australia are driven by high‐pressure weather systems that create southeasterly coastal winds (Gillett & Fyfe, 2013; Grose et al., 2015; IPCC, 2021; O'Grady et al., 2015). The upwelling on the western Tasmanian shelf (Kämpf, 2015), including areas used by shy albatross in our study and year‐round (Hedd & Gales, 2005), is driven by similar wind patterns to the well‐known Bonney upwelling ca. 1000 km west of our study site that supports a large biomass of marine predators in southern Australian waters (Schumann et al., 2013). Under climate change projections, the sub‐tropical ridge (where high‐pressure systems develop) is expected to shift south. This will generate more intense and frequent upwelling in these waters (Gillett & Fyfe, 2013, Grose et al., 2015, IPCC, 2021, O'Grady et al., 2015), which could in fact boost productivity and support a higher biomass of predators in the area (Schumann et al., 2013). With rapid environmental change projected for this region, species like the shy albatross can be sentinels for ecosystem impacts (Hazen et al., 2019; Sydeman et al., 2021).

## AUTHOR CONTRIBUTIONS

**Claire Mason:** Conceptualization (equal); data curation (lead); formal analysis (lead); funding acquisition (equal); investigation (lead); methodology (lead); project administration (lead); resources (equal); visualization (lead); writing – original draft (lead); writing – review and editing (lead). **Alistair J. Hobday:** Conceptualization (equal); data curation (equal); formal analysis (equal); funding acquisition (equal); investigation (equal); methodology (equal); project administration (equal); resources (equal); supervision (equal); visualization (equal); writing – original draft (equal); writing – review and editing (equal). **Mary‐Anne Lea:** Conceptualization (equal); data curation (equal); formal analysis (equal); funding acquisition (equal); investigation (equal); methodology (equal); project administration (equal); resources (equal); supervision (equal); visualization (equal); writing – original draft (equal); writing – review and editing (equal). **Rachael Alderman:** Conceptualization (equal); data curation (equal); formal analysis (equal); funding acquisition (equal); investigation (equal); methodology (equal); project administration (equal); resources (equal); supervision (equal); visualization (equal); writing – original draft (equal); writing – review and editing (equal).

## Data Availability

Data available from Birdlife's Seabird Tracking Database: https://data.seabirdtracking.org/dataset/1381. R Code from: https://github.com/clairemas0n/Shy_incubation_foraging
